# Items

**Published:** 1901-02

**Authors:** 


### ITEMS.

The manufacture of tissue phosphates, carried on for many years by the late Dr. T. P. Wheeler, of Montreal, will be continued as formerly without change of name.

M. J. Breitenbach Co., of New York, well known as the American agents for Gude's pepto-mangan, have again sent out their year-book to the medical profession. The edition for 1901, which is precisely like its predecessors, is one of the most useful desk memorandum books with which we are familiar. Mr. Breitenbach conceived the idea of attaching to each wrapper a sticker advertising the Pan-American Exposition of Buffalo. In this way over 100,000 physicians are reminded at one and the same time of Gude's pepto-mangan and the Pan-American Exposition.

The following letter is self-explanatory:

New York, November 17, 1900.

Mr. Martin H. Smith, 68 Murray St., New York City.

Dear Sir—In reply to your favor of the 13th inst., we would say that we have submitted glyco-heroin to a careful pharmacological investigation. It affords us much pleasure to state that the ingredients of the formula are entirely compatible, and that the heroin is held in permanent solution. It seems to us that you have a very valuable combination of expectorants and sedatives in this formula, and that it will prove a very useful remedy in the class of cases in which it is indicated. Thanking you most cordially for your supply of glyco-heroin, we remain, Yours very truly,

Farbenfabriken of Elberfeld Co.

The post-graduate course in bacteriology, given under the personal supervision of Dr. W. G. Bissell, at the Dental Department U. of B., will begin February 18, 1901, at 8.15 o'clock p.m.

